# The Aortomesenteric Angle as an Aid in Diagnosing Superior Mesenteric Artery Syndrome

**DOI:** 10.5811/cpcem.2016.12.30676

**Published:** 2017-03-16

**Authors:** Kathleen E. Kane, Andrew L. Koons

**Affiliations:** * University of South Florida Morsani College of Medicine, Tampa, Florida; † Lehigh Valley Hospital-Muhlenberg, Department of Emergency Medicine, Allentown, Pennsylvania

## CASE REPORT

We present a case of a 22-year-old female with a history of intravenous drug abuse and homelessness presenting with four days of vomiting and abdominal pain. She noted significant weight loss in the preceding weeks; this was surmised to be secondary to increased drug usage. Examination revealed a diffusely tender but non-peritoneal abdomen. Computed tomography (CT) demonstrated duodenal obstruction with findings consistent with superior mesenteric artery (SMA) syndrome, which presents as high intestinal obstruction. While SMA syndrome can present due to medical conditions such as achalasia, it may also be seen in malnutrition related to substance abuse.

## DISCUSSION

Symptoms are caused by duodenal compression by the narrow angle of the SMA pressing against the posterior structures. The narrow angle of the SMA is due to lack of fat surrounding the vessel.^1^ When paired with appropriate clinical suspicion, diagnosis of SMA syndrome is facilitated by measurement of the angle between the aorta and the SMA by CT or ultrasonography (US). Aortomesenteric (AOM) angles less than 22 to 28 degrees with an AOM distance between 2–8mm are strongly suggestive of SMA syndrome in the correct patient setting (see Image).^2^–^5^ The normal AOM angle is between 45 and 60 degrees, and the normal AOM distance is between 10–20mm.^6^,^7^ As these values drop, the likelihood of SMA syndrome increases. When paired with any one symptom of SMA syndrome (postprandial epigastric pain, anorexia, vomiting, weight loss), the cutoff of 22 degrees has a sensitivity of 42.8% and specificity of 100%; the cutoff of 8mm has a sensitivity and specificity of 100%.^5^ These values can be obtained from a CT or US. Our patient’s CT showed an AOM angle of 21 degrees and AOM distance of 4mm, helping confirm her diagnosis. Treatment is aimed at improving nutrition and treating the underlying cause of weight loss.

## Figures and Tables

**Image:**
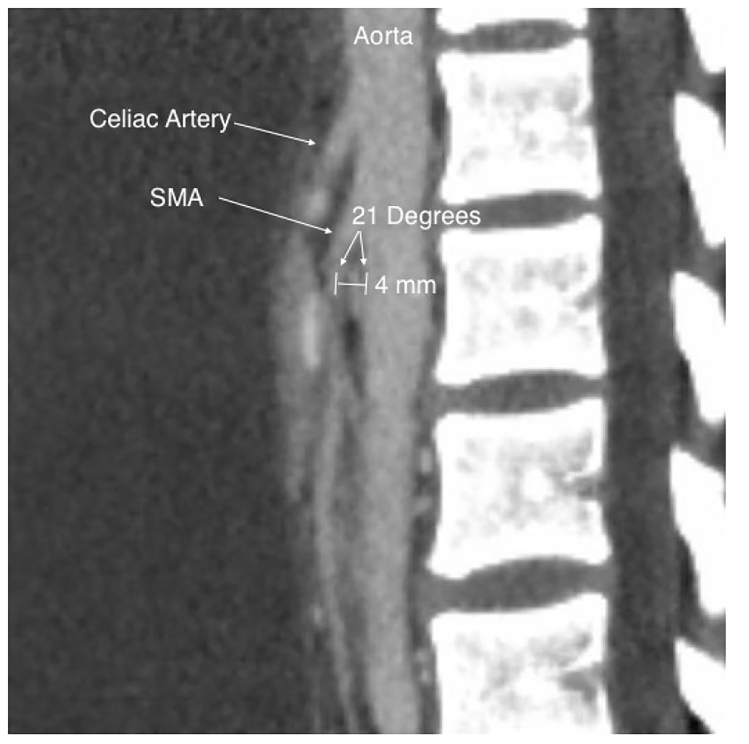
Angle between the aorta and the superior mesenteric artery (SMA) in patient with SMA syndrome.
